# White Matter Microstructure Changes and Cognitive Impairment in the Progression of Chronic Kidney Disease

**DOI:** 10.3389/fnins.2020.559117

**Published:** 2020-09-29

**Authors:** Mengchen Liu, Yunfan Wu, Xixin Wu, Xiaofen Ma, Yi Yin, Huamei Fang, Sihua Huang, Huanhuan Su, Guihua Jiang

**Affiliations:** ^1^The Department of Medical Imaging, Guangdong Second Provincial General Hospital, Guangzhou, China; ^2^The Department of Nephrology, Guangdong Second Provincial General Hospital, Guangzhou, China

**Keywords:** chronic kidney disease, white matter, tract-based spatial statistics, cognitive function, calcium

## Abstract

**Background:**

Cognitive impairment is a well-defined complication of chronic kidney disease (CKD), but the neural mechanisms are largely unknown.

**Objectives:**

The study aimed to assess white matter (WM) microstructure changes and their relationship with cognitive impairment development during CKD progression.

**Methods:**

Diffusion tensor imaging (DTI) datasets were acquired from 38 patients with CKD (19 patients were at stage 3; 19 patients were at stage 4) and 22 healthy controls (HCs). Tract-based spatial statistics (TBSS) was implemented to assess the differences in WM integrity among the three groups. The associations between abnormal WM integrity and clinical indicators (digit symbol test scores, the type A number connection test scores, hemoglobin, serum urea, serum creatinine, serum calcium, and serum potassium levels) were also computed.

**Results:**

Compared with patients with CKD at stage 3 and HCs, patients with CKD at stage 4 showed significantly lower fractional anisotropy (FA) and higher mean diffusivity (MD) in the corpus callosum (CC), anterior thalamic radiation, inferior fronto-occipital fasciculus, and inferior longitudinal fasciculus. Correlation analysis showed that the MD in the genu of CC was negatively associated with the digit symbol test scores (*r* = -0.61, *p* = 0.01), and the FA in the left anterior thalamic radiation was positively associated with the level of serum calcium (*r* = 0.58, *p* = 0.01).

**Conclusion:**

Patients with non-end-stage CKD have multiple abnormalities in WM regions. DTI metrics change with the progression of CKD and are primarily associated with cognitive impairment. The reduced integrity of WM tracts may be related to a low level of blood calcium.

## Introduction

Chronic kidney disease (CKD) is defined as a glomerular filtration rate of less than 60 ml/min/1.73 m^2^ or increased urinary albumin excretion for no less than 3 months. The current study suggests that one in 10 people will develop CKD at some point in their life (Jha et al., 2013). Cognitive impairment is a well-defined complication of CKD (Bugnicourt et al., 2013). Impaired cognitive function may lead to the progression of kidney disease due to difficulties in adhering to the CKD risk reduction strategies (Kurella Tamura et al., 2016). By using cognitive function testing, studies have found that patients with stage 3 or 4 CKD have impaired processing speed, short-term memory, recall memory, and sequencing function compared with healthy people (Berger et al., 2016), which suggests that neurocognitive deficits are already present in the non-end-stage of CKD. However, so far, brain imaging studies of patients with non-end-stage kidney disease are limited. Recent structural MRI studies have found abnormal brain volumes in patients with CKD (Murea et al., 2015; Freedman et al., 2017; Hartung et al., 2018), but it remains unclear whether specific brain changes associated with cognitive impairment will occur in the non-end-stage of CKD.

DTI is a non-invasive neuroimaging technique that has been used to detect changes in white matter (WM) microstructural properties in recent years (Le Bihan, 2003; Hagmann et al., 2006). Tract-based spatial statistics (TBSS) and voxel-based analysis studies have found that patients with CKD have abnormal WM integrity in the corpus callosum (CC) (Chou et al., 2013), fronto-temporal connections (Drew et al., 2017), and anterior thalamic radiation (Yin et al., 2018). However, these studies included patients with hemodialysis as subjects; long-term hemodialysis exerts gradual damage to the integrity of WM (Kong et al., 2014; Drew et al., 2017; Eldehni et al., 2019), so the impact of CKD itself on WM microstructural properties remains unclear. TBSS is a method for analyzing WM fiber bundles that have been shown to improve the sensitivity of detecting WM diffusion changes, even for a relatively small sample size (Focke et al., 2008; Yeh et al., 2009). In one study by Mina (2018) TBSS analysis was used to investigate the WM microstructural changes in children with CKD. They found that the anterior limb of the internal capsule had abnormal myelination (Matsuda-Abedini et al., 2018). However, the subject group included renal transplant recipients and peritoneal dialysis patients, so the researchers could not completely rule out the effects of peritoneal dialysis and kidney transplantation on the results.

Our hypothesis is that the WM structure changes are already present in non-end-stage CKD and that these changes are associated with cognitive impairments.

## Materials and Methods

### Participants

We recruited 38 patients diagnosed with CKD (19 patients were at stage 3; 19 patients were at stage 4). The stage of CKD was defined based on the K/DOQI classification for CKD, with 30–59 and 15–29 ml/min/1.73 m^2^ classified as stage 3 and 4 CKD, respectively, and estimated glomerular filtration rate was calculated using Tc-99mDTPA-based single-photon emission computed tomography (Kang et al., 2017). Eligible participants were aged 20–65 years, and their renal function had been stable for at least 3 months. Patients with factors that may affect outcomes such as dialysis and kidney transplantation were excluded from the study. Healthy controls (HCs) were also recruited individuals with similar age, sex, and education level to the participants with CKD. The exclusion criteria were a history of drug or alcohol abuse, significant neurological disorders, psychiatric disorders, and traumatic brain injury. The project was approved by the Research Ethics Review Board of the Institute of Mental Health at our hospital. Written informed consent was obtained from all the participants. HCs have similar age, sex, and education level with the participants with CKD.

### Neuropsychological Tests

All patients underwent multiple neuropsychological tests before MR data acquisition, including the digit symbol test based on Webster’s Adult Intelligence Scale, the type A number connection test (i.e., part A of the Trail Making Test), the Chinese revision of the Folstein version of the Mini-Mental State Examination, and Beijing revised version of Montreal Cognitive Assessment (MoCA). MoCA is a fast cognitive assessment tool that has proven to be effective in the CKD population. MMSE is a comprehensive assessment of cognitive function. NCT-A and digit symbol test mainly reflect information processing speed and attention.

### Laboratory Tests

Several biochemical tests (hemoglobin, serum urea, serum creatinine, serum calcium, and serum potassium levels) were measured before MR data acquisition. No blood laboratory test was performed on the participants in the control group.

### Image Acquisition and Processing

All MRI scans were performed using a Philips Ingenia 3.0T scanner with a 32-channel phased-array head coil. DTI data were acquired using a sensitivity-encoding parallel single-shot echo-planar imaging (SENSE-EPI, reduction factor = 2) sequence; the parameters were as follows: TR/TE = 3,870/86 ms, voxel size = 2 mm × 2 mm × 2 mm, matrix = 112 × 109, FOV = 224 mm × 224 mm, slice thickness = 2 mm, without a slice gap. The DTI scheme included 64 non-collinear diffusion gradient directions (*b* = 1,000 s/mm^2^) and one reference image (*b* = 0 s/mm^2^). A total of 66 axial slices were collected, covering the whole brain, and the duration of the DTI scan was 4 m and 20 s.

DTI data were preprocessed using the FMRIB Software Library-based PANDA toolbox. Fractional anisotropy (FA) and mean diffusivity (MD) maps were calculated using TBSS automatically after all DTI data were corrected for image distortions due to eddy current artifacts and subject motion effects. Voxel-wise analysis was performed using TBSS. The FA maps of all participants were placed into a standard space for non-linear registration, and then the most representative FA image was identified as the common space. The mean FA image was then created and thinned to create a mean FA skeleton that represented the centers of all tracts common to all participants (FA threshold > 0.2). Next, the aligned FA data from each subject were mapped onto this skeleton. MD maps were also mapped onto the mean FA skeleton using the non-linear registrations of the FA images. ANOVA was performed on the differences in FA and MD among the three groups using the randomized statistical analysis panel implemented in FMRIB Software Library. Five thousand random permutation-based non-parametric permutation inference and threshold-free cluster enhancement (TFCE) correction method were used to perform voxel analysis on multiple comparisons. The TFCE results were corrected to a *P*-value of less than 0.05 to enable the identification of differences among groups. The Johns Hopkins University White Matter Tractography Atlas was added in FMRIB Software Library to describe the localization of all the anatomical clusters.

### Statistical Analysis

All statistical calculations were performed with IBM SPSS software, version 20.0. The sample mean comparisons were performed using a two-sample *t*-test or one-way ANOVA, expressed as mean ± standard deviation, and the categorical data were analyzed by χ^2^ test; non-normal distribution data were analyzed by Kruskal–Wallis *H* test, expressed as the median and the interquartile range. Partial correlation analysis was used to assess correlations between the mean FA, MD values from clusters with significant group differences, and the clinical indicators (digit symbol test scores, the type A number connection test scores, hemoglobin, serum urea, serum creatinine, serum calcium, and serum potassium levels), with age and education level as covariates. A *p*-value less than 0.05 was defined as a significant difference.

## Results

### Demographic and Clinical Features

The demographic characteristics and the clinical data of the CKD patients and HCs are provided in Table 1. The participants with CKD and the controls were similar with respect to age, sex, and education. Compared with the patients with CKD3 and HCs, the Mini-Mental State Examination, the MoCA, and the digit symbol test scores in the CKD4 group were significantly lower.

**TABLE 1:** Demographics, neuropsychological test scores, and biochemical parameters of CKD3 and CKD4 patients and healthy controls.

	Healthy controls (*n* = 22)	CKD (*n* = 38)		*P*-value	CKD3 vs. CKD4	HC vs. CKD3	HC vs. CKD4
		
		CKD3 (*n* = 19)	CKD4 (*n* = 19)				
Age (y)	43.72 ± 10.66	45.00 ± 14.31	47.53 ± 11.35	0.537			
Sex (male/female)	14/8	14/5	11/8	0.586			
Education (years)	9.45 ± 2.79	8.53 ± 2.63	8.00 ± 3.23	0.270			
**Neuropsychological tests**							
MoCA score	28.50 (25.00, 30.00)	25.00 (22.00, 29.00)	24.00 (19.00, 28.00)	0.005	1.000	0.062	0.006
MMSE score	30.00 (28.75, 30.00)	29.00 (27.00, 30.00)	28.00 (26.00, 29.00)	0.012	0.313	0.591	0.009
NCT-A score	39.50 (33.75, 45.50)	45.00 (37.00, 71.00)	47.00 (32.00, 83.00)	0.200			
DST score	47.00 (42.75, 59.25)	37.00 (18.00, 56.00)	34.00 (17.00, 49.00)	0.016	1.000	0.104	0.020
**Biochemical parameters**							
Hemoglobin (g/dl)		114.16 ± 20.29	101.42 ± 17.61	0.046			
Urea nitrogen (mg/dl)		11.37 ± 3.96	14.98 ± 5.43	0.025			
Calcium (mg/dl)		2.30 ± 0.14	2.14 ± 0.22	0.10			
Creatinine (mg/dl)		272.16 ± 106.58	437.95 ± 168.44	0.001			
Kalium (mg/dl)		4.10 ± 0.41	4.58 ± 0.54	0.004			
eGFR (ml/min/1.73 m^2^)		38.96 ± 7.63	21.04 ± 4.50	< 0.001			

*MoCA, Montreal Cognitive Assessment; MMSE, Mini-Mental State Examination; NCT-A, type A number connection test; DST, digit symbol test.*

### TBSS Analysis Between Groups

#### Between the CKD3 and CKD4 Groups

**FIGURE 1:**
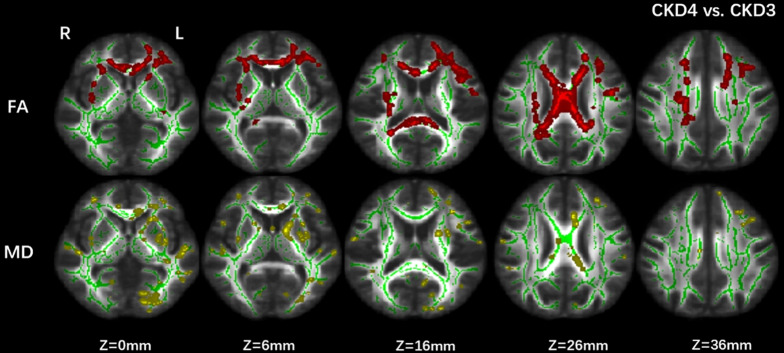
Group differences between CKD3 and CKD4. Tract-based spatial statistics analysis of (FA) and (MD) volumes revealed lower FA values (in red) and higher MD values (in yellow) in CKD4 group when compared with CKD3 group. Results are superimposed on fiber skeleton (green) and overlaid on the FMRIB FA 1-mm template. The threshold for the results was set at *P* < 0.05 (threshold-free cluster enhancement corrected). FA, fractional anisotropy; MD, mean diffusivity; L, left; R, right.

The CKD4 group showed significantly lower FA and higher MD values (*p* corrected < 0.05) in the CC, superior longitudinal fasciculus, and inferior fronto-occipital fasciculus than the CKD3 group (Figure 1).

#### Between the CKD4 and Control Groups

**FIGURE 2:**
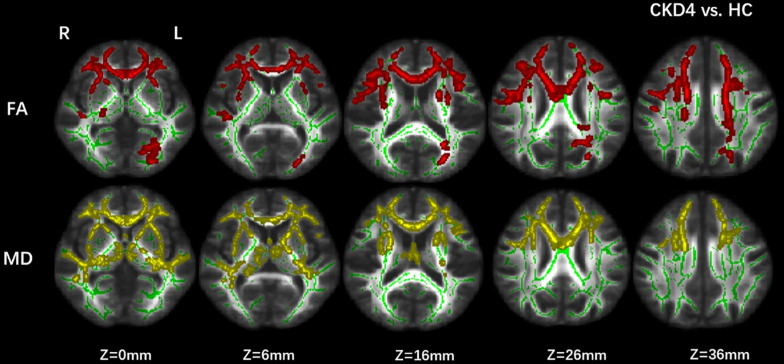
Group differences between CKD4 and healthy controls. Tract-based spatial statistics analysis of FA and MD volumes revealed lower FA values (in red) and higher MD values (in yellow) in CKD4 group when compared with healthy controls. Results are superimposed on fiber skeleton (green) and overlaid on the FMRIB FA 1-mm template. The threshold for the results was set at *P* < 0.05 (threshold-free cluster enhancement corrected). FA, fractional anisotropy; MD, mean diffusivity; L, left; R, right.

Decreased FA in CKD4 (*p* corrected < 0.05) was found in the superior longitudinal fasciculus, the body and genu of the CC, inferior fronto-occipital fasciculus, inferior longitudinal fasciculus, and anterior thalamic radiation compared with the control group. Moreover, increased MD (*p* corrected < 0.05) was found in the CC, anterior thalamic radiation, inferior fronto-occipital fasciculus, and inferior longitudinal fasciculus in the CKD4 group (Figure 2).

#### Between the CKD3 and Control Groups

The differences between CKD3 and HCs did not meet the stringent statistical threshold of our study (TFCE corrected *p* < 0.05), but differences between CKD3 and HCs were found at a liberal threshold (*p* < 0.05, uncorrected).

### Correlation Analysis

**FIGURE 3:**
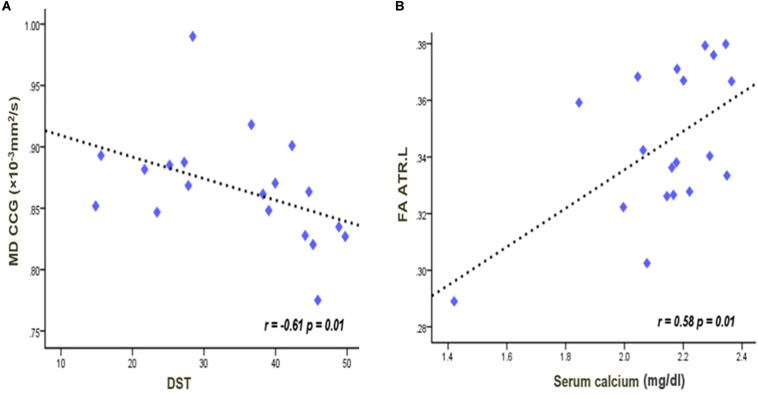
Significant negative correlations between the MD values in the genu of the corpus callosum and digit symbol scores **(A)**. Significant positive correlations between the serum calcium and the FA values in the left anterior thalamus radiation **(B)**. The lines were linearly fitted from these measurements. *r* values are correlation coefficients of the diffusion tensor imaging parameter and clinical indicators. FA, fractional anisotropy; MD, mean diffusivity; CCG, corpus callosum genu; ATR, anterior thalamus radiation; DST, digit symbol test.

In the CKD4 sample, the MD values in the genu of CC were negatively correlated with digit symbol test scores (*r* = −0.61, *p* = 0.01) (Figure 3). In the left anterior thalamus radiation, the FA value was positively correlated with the serum calcium concentration (*r* = 0.58, *p* = 0.01) (Figure 3). Correlations were not significant between the other indicators.

## Discussion

Our study used the TBSS method to characterize the WM microstructure changes on cognitive impairment in patients with non-end-stage CKD. Compared to HCs, the CKD 4 group showed decreased FA and increased MD in the body and genu of the CC, anterior thalamic radiation, inferior fronto-occipital fasciculus, and inferior longitudinal fasciculus. Decreased FA values in the CC, superior longitudinal fasciculus, inferior fronto-occipital fasciculus, and inferior longitudinal fasciculus were also found in the CKD4 group compared with the CKD3 group. The MoCA, Mini-Mental State Examination, and digit symbol test of the CKD4 group were lower than HCs. The MD value in the genu of the corpus callosum was significant negatively correlated with the scores of digit symbol test in the CKD4 group. These results indicate associations between WM microstructure changes and specific cognitive functions, supporting the hypothesis that brain microstructure abnormalities affect cognitive impairment during the progression of CKD. In addition, the FA value in the left anterior thalamic radiation was positively associated with the level of serum calcium. Because only stages 3 and 4 CKD patients were included in our study, we ruled out the effects of hemodialysis and kidney transplantation on the results. Our study reveals the impact of CKD itself on WM microstructure changes; this is the first batch of studies on WM neurobiological changes in non-end-stage CKD with TBSS analysis.

In this study, WM abnormalities were most significant in patients with CKD at stage 4, mainly located in the CC, inferior fronto-occipital fasciculus, inferior longitudinal fasciculus, and superior longitudinal fasciculus. By comparison, a recent TBSS study investigated the association between cognitive impairment and WM integrity in patients with end-stage CKD and found decreased FA values in the corona radiata, the anterior thalamic radiation, the inferior fronto-occipital fasciculus, the body and genu of the CC, the superior longitudinal fasciculus, and the inferior longitudinal fasciculus in patients with end-stage CKD (Yin et al., 2018). Inconsistent with previous studies on WM integrity in ESRD patients, our study reported fewer WM deterioration areas, which might be attributable to the fact that the subjects in our study are at CKD stage 4, which further supports the hypothesis that the severity of WM deterioration might be related to the stage of CKD. These results were supported by previous studies using various tasks to assess cognitive impairment in CKD (Nasser et al., 2012; Egbi et al., 2015). These studies indicated that cognitive impairment exhibits a cumulative effect on the stages of CKD (Brodski et al., 2018). Our study observed the difference in WM integrity between CKD3 and CKD4 patients but did not find significant differences in the scores of neuropsychological tests on CKD3 and CKD4 groups, probably because the limited cognitive tests used in this study only provide partial changes in cognitive function, which made the study of cognitive function limited. Further longitudinal studies are necessary to assess this possibility.

While DTI research on patients with non-end-stage CKD remains sparse, many abnormalities in the WM region reported in this study have previously been reported in DTI studies using end-stage kidney disease samples. For example, most DTI studies on end-stage kidney disease have found decreased FA in the CC (Gupta et al., 2016; Yin et al., 2018). The CC is the largest WM structure of the human brain, and it participates in interhemispheric information integration. Reduced WM integrity in the CC will reduce the connection between the cerebral hemispheres, thereby reducing long-distance interaction control of motion, perception, and cognition (Doron and Gazzaniga, 2008; Glickstein and Berlucchi, 2008). In our study, the CKD4 group had lower scores on NCT-A and DST tests than the HCs, suggesting that the information processing speed function of CKD4 patients was damaged. In addition, the results of a correlation analysis showed that the MD value of the genu of the corpus callosum was negatively correlated with the scores of digit symbol test, suggesting that the injury of the corpus callosum in CKD4 patients might be related to the impairment of information processing speed. This finding is consistent with a previous study. By using DTI and magnetization transfer imaging, Kourtidou et al. (2013) investigate WM alterations in the CC in patients with severe traumatic brain injury, and digit symbol test performance was related to CC FA, which demonstrate that significant alterations in WM are related to information processing speed. It means that the CC likely plays an important role in CKD cognitive dysfunction.

Our findings also showed degeneration of the inferior fronto-occipital fasciculus, inferior longitudinal fasciculus, and superior longitudinal fasciculus in the CKD4 group compared to the CKD3 group and HCs. The inferior fronto-occipital fasciculus is an important association pathway between the frontal, temporal, and occipital lobes and affects neuromotor function and visual and auditory processing (Kvickstrom et al., 2011). The inferior longitudinal fasciculus is a critical connection from the inferior occipital gyrus to the temporal lobe (Gold et al., 2012). Damaged inferior longitudinal fasciculus and superior longitudinal fasciculus integrity are associated with thought disorders and cognitive impairments (Makris et al., 2005; Haghshomar et al., 2018). We also found reduced FA values in the anterior thalamic radiation when we compared the CKD4 group with HCs, consistent with the results of previous studies. Yin et al. (2018) used TBSS analysis in investigating patients with end-stage CKD and found WM deterioration in the patients’ anterior thalamic radiation tracts. Studies have found that cognitive decline in patients with Alzheimer’s disease is associated with impaired integrity of the thalamic connection (Zhu et al., 2015). Previous studies have also shown that the anterior thalamic radiation is associated with executive function (Watanabe and Funahashi, 2012) and memory coding (Mamiya et al., 2018), which can increase cross-domain learning. Previous findings indicate that the anterior thalamic radiation might play a role in executive function and memory *via* a neuropathological mechanism, including that of CKD.

Our results supported our hypothesis that the effects of more severe CKD may lead to more serious WM damage, and WM damage is associated with cognitive impairment. This finding is supported by a previous study using a six-item cognitive impairment test, which found that CKD4 patients had significantly worse cognitive abilities than CKD3 patients, almost double the memory and attention deficits (Egbi et al., 2015). This suggests an association between renal dysfunction and the prevalence of cognitive impairment in CKD, but the authors did not discuss the potential reasons for this finding. Our study found a positive correlation between the FA values in the left anterior thalamic radiation and the serum calcium concentration in the CKD4 group. One previous study used magnetic resonance diffusion-weighted imaging to identify WM deterioration associated with rotavirus-induced hypocalcemia in neonates. A possible explanation is that hypocalcemia may cause neurotoxicity and neurotransmitter imbalance (Ku et al., 2018), but little is known about how calcium metabolism affects cognition in CKD patients. Notably, hypocalcemia can directly stimulate the production of parathyroid hormone, leading to excessive mobilization of calcium from the bone to maintain normal serum calcium levels (Ritz et al., 1995). Excessive parathyroid hormone is considered neurotoxic and results in cognitive impairment; high parathyroid hormone increases calcium influx in the brain, affecting the neurotransmission of the central nervous system and leading to neurotoxicity (Slatopolsky et al., 1980; Wilmskoetter et al., 2019). However, at stage 3 of CKD, calcium metabolism disorder is not serious, and thus WM deterioration occurs later in CKD. Significant differences between CKD3 and HCs were found only at a liberal threshold and may also have been affected by the relatively small sample size.

Our study has several limitations. The small size of our research sample may limit the universality of the results. Further longitudinal studies of larger samples are needed to determine the effect of CKD on WM. The neurocognitive test scale used in our research is insufficient, resulting in low sensitivity to cognitive function assessment in different areas of CKD patients. Additionally, we did not rule out the effects of blood pressure, which may affect the reversibility of cognitive function. Future research should include more relevant clinical indicators and neurocognitive assessments to clarify their correlation with microstructural changes.

## Conclusion

In conclusion, we found that patients with CKD4 showed significant WM deterioration, compared to patients with CKD3 and HCs, by means of fractional anisotropy and mean diffusivity. This finding confirms the relationship between abnormalities of WM microstructure and cognitive impairment in CKD; this anomaly may be related to low blood calcium. WM deterioration appears in patients with CKD as the diseases progress. These findings provide valuable insights into the relationship between WM microstructural changes and cognitive impairment as well as the possible neurophysiological mechanisms in patients with non-end-stage CKD.

## Data Availability Statement

The raw data supporting the conclusions of this article will be made available by the authors, without undue reservation.

## Ethics Statement

The studies involving human participants were reviewed and approved by the Ethics Committee of Guangdong Second Provincial General Hospital. The patients/participants provided their written informed consent to participate in this study.

## Author Contributions

ML designed the experiment. ML, YW, and XW carried out the experiment. XM and YY collected and sorted out the data. HF, SH, and HS helped on data management and processing. ML, YW, XW, and GJ wrote the manuscript. All authors contributed to the article and approved the submitted version.

## Conflict of Interest

The authors declare that the research was conducted in the absence of any commercial or financial relationships that could be construed as a potential conflict of interest.
